# Full-Spectrum Analysis of Perovskite-Based Surface Plasmon Nanolasers

**DOI:** 10.1186/s11671-020-3290-6

**Published:** 2020-03-29

**Authors:** Pi-Ju Cheng, Qi-Yan Zheng, Chu-Yuan Hsu, Heng Li, Kuo-Bin Hong, Yizhi Zhu, Qiannan Cui, Chunxiang Xu, Tien-Chang Lu, Tzy-Rong Lin

**Affiliations:** 1 Research Center for Applied Sciences, Academia Sinica, Taipei, 11529 Taiwan; 2 Department of Mechanical and Mechatronic Engineering, National Taiwan Ocean University, Keelung, 20224 Taiwan; 3 Department of Photonics, National Chiao Tung University, Hsinchu, 30010 Taiwan; 4 State Key Laboratory of Bioelectronics, School of Biological Science and Medical Engineering, Southeast University, Nanjing, 210096 China; 5 Institute of Optoelectronic Sciences, National Taiwan Ocean University, Keelung, 20224 Taiwan; 6 Center of Excellence for Ocean Engineering, National Taiwan Ocean University, Keelung, 20224 Taiwan

**Keywords:** Perovskites, Surface Plasmons, Nanolasers, Nanowires

## Abstract

We systematically studied the characteristics of hybrid perovskite-based surface plasmon nanolasers. If one changes the anion composition of perovskites, the emission wavelength can be easily tuned. We conducted in full-spectrum modeling that featured hybrid perovskite nanowires placed on different SiO_2_-coated metallic (Au, Ag, and Al) plates. The proposed nanocavities that supported plasmonic gap modes exhibited distinguished properties of nanolasers, such as low-transparency threshold-gain and low lasing threshold. The corresponding experimental results for the MAPbBr_3_ nanolaser on Ag revealed the low-threshold operation. These superior features were attributed to enhanced light-matter interaction with strong coupling. Therefore, the proposed scheme, integrated with hybrid perovskite as gain material, provides an excellent platform for nanoscale plasmon lasing in the visible to near-infrared spectra.

## Introduction

Methyl ammonium lead halide perovskites MAPbX_3_, (MA = CH_3_NH_3_, X = I, Br, Cl), a class of hybrid organic-inorganic semiconductors, exhibit excellent optical properties suitable for semiconductor lasers because of their low non-radiative recombination rates and long carrier lifetimes [1]. Additionally, mixed halide hybrid perovskites can achieve broad energy bandgap tunability corresponding to emitting wavelengths covering the visible and parts of near-infrared spectra regions [2–4]. Several perovskites have been proven to be efficient optical gain materials, for example, in the form of thin films, nanoplates, and nanocrystals [2, 4–10]. However, the high lasing threshold is a concern in the use of perovskites in practical applications such as electrical-driven lasing [11] or optoelectronic integration systems. Their high crystalline quality (single crystal) may diminish the scattering loss [12] and lower the threshold during the pumping process. Recently, solution-processable perovskite nanowires (NWs) have been successfully demonstrated [1]. With two end facets as reflectors, the perovskite NWs naturally form a miniaturized optical cavity. The benefits, in addition to their remarkable electrical properties because of their strong intrinsic exciton oscillation strength, render perovskite NWs an excellent platform for realizing miniaturized devices such as room-temperature, low-cost and low-threshold, exciton-polariton lasers in a compact size [6, 13–16].

However, the footprint of optical modes associated with NW cavities is restricted by the diffraction limit. Surface plasmon polaritons (SPP) have been used to minimize the feature sizes of electromagnetic modes [17, 18]. Various plasmonic NW cavities have been investigated recently [19–23]. Cavities in a metal-insulator-semiconductor scheme are especially promising for sustaining hybrid plasmonic gap modes [24–28]. Therefore, we placed samples of doped or pure perovskite NWs on insulator-coated metallic plates to form plasmonic Fabry-Perot cavities. The resonant modes, resulting from circulation along the NW long axes of the plasmonic gap guided modes, are highly confined by the NWs. The reduced effective modal volume can boost the local photon density of states and the coupling strength between the excitons and photons. The laser characteristics of the proposed nanolaser as a robust cavity for lasing were investigated in this study. For example, the end facets of NWs may not be sufficient to reflect the guided plasmonic gap mode as mirrors, which may increase the threshold gain of cavities drastically. Additionally, intensive research interests are the capacities of common plasmonic metals such as gold (Au), silver (Ag), aluminum (Al), or copper (Cu) to lower the modal volume efficiently without any deterioration of laser performance throughout the visible to the near-infrared wavelength spectrum region.

**Fig. 1:**
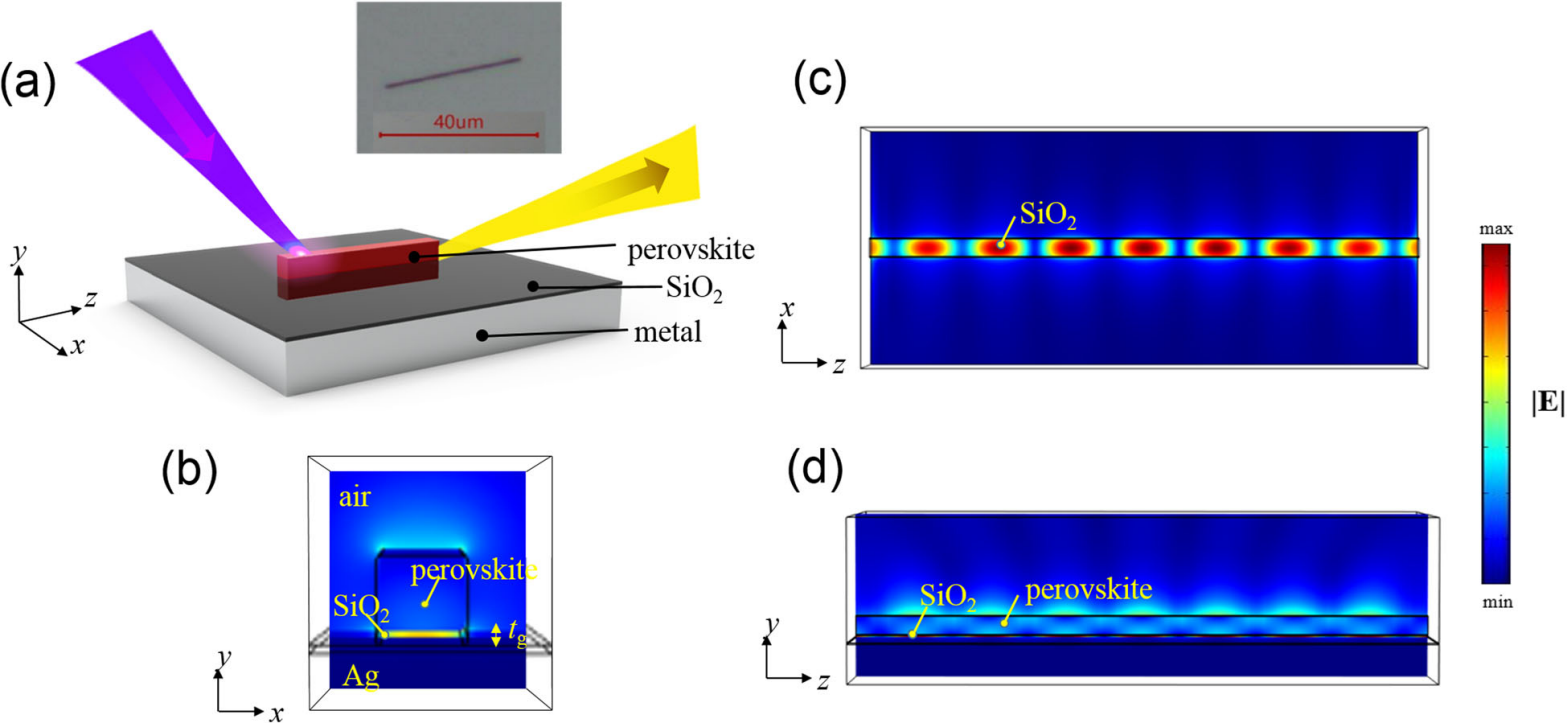
Plasmonic perovskite nanocavity. **a** Schematic diagram of proposed plasmonic nanocavity. A perovskite nanowire is placed on a SiO_2_-covered metallic substrate. Two end facets of the nanowire with a length of several micrometers, which function as reflectors, form naturally a plasmonic cavity. The inset is an optical microscopy picture of a MAPbBr_3_ NW on a SiO_2_-covered Ag substrate. **b–d** Modal profiles (in transverse view) of electric field component |**E**| of the cavity resonance mode calculated by 3D finite element method. The strongly confined modal profile of plasmonic gap mode is shown in (**b**). The resonance pattern shown in (**d**) depicts the features of the hybrid plasmonic mode originated from the coupling of NW photonic mode and propagating surface plasmon wave. In addition to an obvious standing-wave pattern along the long axis (*z*-direction) as shown in (**c**), lateral confinement of the mode (*x*-direction) is sufficiently strong

In this study, we analyzed the characteristics of perovskite-based nanolasers placed on different SiO_2_-coated metallic (Au, Ag, and Al) plates over a wide spectrum by using the finite-element method (FEM: COMSOL package [29]). For single-crystal pure perovskite MAPbX_3_, the spectral gain windows related to the band transitions in the first Brillouin zone for X = Cl, Br, I are approximately 2.9 eV, 2.2 eV, and 1.5–1.6 eV [30], respectively, with corresponding emitting wavelengths *λ* = 425, 555, and 800 nm. The NWs depicted in the inset of Fig. 1a illustrating active region in the proposed nanocavities exhibit smooth surface morphology which can reduce the scattering loss during lasing. By converting the perovskites into those doped with a different halogen anion using the ion exchange reaction method [31], we can expand the emitting spectrum of perovskites to the nearly fully visible wavelength region. Of all the plasmonic metals, Ag exhibits a relatively low metal loss in the visible wavelength region, and Al, being a low-cost element, receives considerable attention because of its excellent plasmonic properties in the blue to ultraviolet wavelength region [32]. Au is commonly considered suitable for plasmon wave generation at the infrared region. These three metals are selected to be plasmonic media to enhance the charge-photon interaction in the system.

First, we investigated the modal features of the fundamental hybrid plasmonic guided modes on SiO_2_/Ag, SiO_2_/Al, and SiO_2_/Au metal plates using the two-dimensional (2D) FEM. Hybrid plasmonic gap modes originate from the coupling between the photonic and surface plasmon modes at the insulator-metal interface. Strong coupling strengths may result in severe intrinsic material loss because of the large overlap of modes with metal, which considerably depends on the gap thickness *t*_g_. We accordingly solved the modal loss, modal profiles, confinement factors, and transparency threshold gains of the hybrid plasmonic gap modes at various gap thickness *t*_g_, as indicated in Fig. 1b. The width of NWs was set to 100 nm at the cavity length *L* of 2.67 μm, which was comparable with the NWs obtained using the self-assembly method [33, 34]. Subsequently, the calculations of the resonant modes in nanocavities are implemented with the three-dimensional (3D) FEM [29]. Empirical calculations have proven that Ag is the best metal for MAPbBr_3_ nanolaser.

Therefore, we developed a low-threshold MAPbBr_3_ nanolaser on a SiO_2_-covered silver substrate through optical pumping. The proposed nanolaser exhibited an extremely small modal footprint, low lasing threshold, and tunable emission wavelengths, which can be used in applications such as next-generation light sources in the future.

## Method

### Preparation of Perovskite Nanowire Cavities

Because Ag exhibited the best plasmonic characteristics in nanolaser operations, we used MAPbBr_3_ NWs on the Ag plate with a 10-nm-thick SiO_2_ as a spacer layer to investigate nanolaser performance. The Ag plate was prepared using an e-gun evaporator on the Si substrate; the growth and annealing parameters were optimized for flat surface roughness followed by the deposition of the SiO_2_ layer [35]. MAPbBr_3_ NW synthesis was based on the one-step solution self-assembly method [33, 34]. First, 0.15 mmol MABr and 0.15 mmol PbBr_2_ powders were dissolved in 5 ml N, N-dimethylformamide, which functioned as the precursor solution. The precursor solution was then drop-casted on SiO_2_-covered Ag plates. Second, the substrate supporting Ag plates was placed on a stage in a beaker containing dichloromethane. The substrate was approximately 3 cm above the liquid surface of dichloromethane. Finally, the beaker covered by one layer of aluminum foil was placed in an incubator at 60 °C. In 4 h, the evaporation process of liquids in the beaker was accomplished and MAPbBr_3_ NWs were obtained on SiO_2_-covered Ag plates. We then mounted the NW nano-cavities, with configurations shown in Fig. 1a, in a high-vacuum chamber at 77 K.

### Characterization of Lasing Action

To investigate the lasing action of a single NW cavity, we used the scanning electron microscope to search for MAPbBr_3_ NWs with a width approximately 100 nm and length closed to 3 μm. After identifying the location of those NWs, the samples were placed in a cryo-chamber for optical pumping. A third harmonic generation of a Nd:YVO_4_ pulse laser emitted at 355 nm was used as the pumping source, and the pulse duration and the repetition rate were 0.5 ns and 1 kHz, respectively. A × 100 near-ultraviolet infinity-corrected objective lens with a numerical aperture of 0.5 (Mitutoyo) was applied to focus the laser beam to the MAPbBr_3_ NW with the focal spot size approximately 15 μm in diameter. Only one NW was pumped at a time. Then, the emission signal of MAPbBr_3_ NW was collected using the same objective lens. An optical fiber with a 600-μm core diameter was attached to the lens. To collect the output emission from the end mirrors of NWs at various frequencies, a nitrogen-cooled charge coupled device was attached to a 320-mm-long single monochromator (iHR320, Horiba) at the other end of the fiber.

## Results and Discussion

The proposed nanocavity exhibits low-threshold and strong modal confinement, depicted in Fig. 1a. We determined the resonance modes to investigate the characteristics of the cavity. The modal profiles of the nanocavity featuring a perovskite NW on a SiO_2_/Ag plate are presented in Fig. 1. We proved that the transverse views of the resonance mode profile |**E**| (b) at an antinode of the profile along the *z*-axis (*x*-*y* plane), (c) in the middle of thin gap (below the NW) (*x*-*z* plane), and (d) by bisecting the NW (*y*-*z* plane), respectively. As depicted in Fig. 1b, the profile of the cavity mode is indeed strongly confined with the features of the guided hybrid gap mode. The resonance pattern illustrated in Fig. 1d reveals the characteristics of both the NW photonic leaky modes (width below cutoff dimension) and propagating surface plasmon waves. In addition to an obvious standing-wave pattern along the long axis (*z*-direction) depicted in Fig. 1c, the lateral distribution of the mode (along the *x*-direction) defined by the small NW of nanoscale width is also sufficiently confined, which concurs with the plasmonic mode characteristics.

### Characteristics of Plasmonic Hybrid Perovskite Waveguides

**Fig 2:**
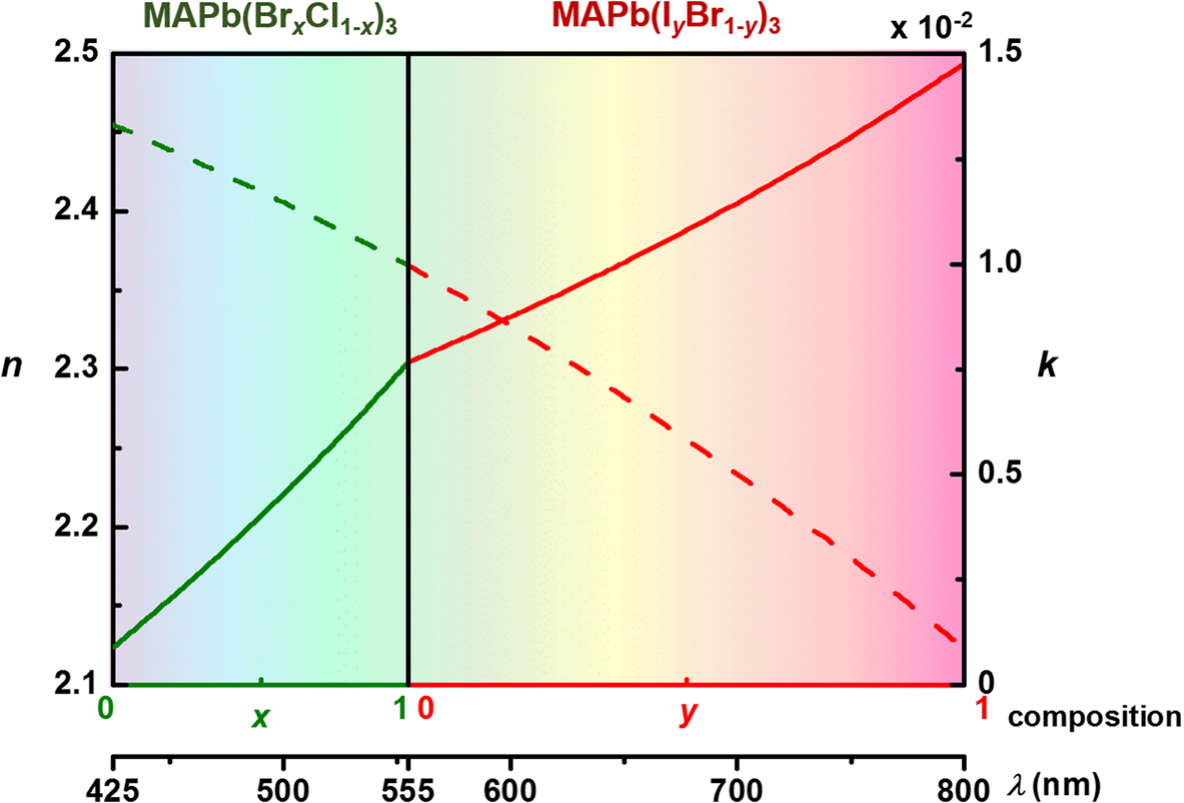
Dispersive properties of compositional hybrid MAPbX_3_. Complex refractive indices (*n*, *k*) of hybrid perovskites MAPb(Br_*x*_Cl_1-*x*_)_3_ (green lines) and MAPb(I_*y*_Br_1-*y*_)_3_ (red lines) of various compositions (*x* and *y*) emitting at wavelengths over the visible and infrared spectrum. Refractive indices of pure perovskites MAPbCl_3_, MAPbBr_3_, and MAPbI_3_ at compositions *x* = 0, *x* = 1 (*y* = 0) and *y* = 1 are (2.2, 0.013), (2.30, 0.01), and (2.49, 0.0009). They are emitting at wavelength *λ* = 425, 555 and 800 nm

To investigate the plasmonic lasing characteristics in the visible to near-infrared wavelength region, the dielectric function of the hybrid version of Br-doped MAPbCl_3_ (MAPb(Br_*x*_Cl_1-*x*_)_3_) and I-doped MAPbBr_3_ (MAPb(I_*y*_Br_1-*y*_)_3_) were examined. In the single-crystal perovskite MAPbX_3_, complex electronic configurations originate from the hybridization of an organic group, lead cationic, and halogen anionic states which causes multiple electronic transitions. In the lattice of doped MAPbX_3_, the dopants and vacancies, introduced during ion exchange reaction, may lower the crystalline quality and smear discrete electronic states. Therefore, instead of performing rigorous first-principle band calculations [36] to reveal each distinct absorption peak on the dispersion relation of the dielectric function, we denote that dielectric function *ϵ* as a simple function of the emission energy bandgap (*E*_*g*_) of the mixed perovskites (MAPb(Br_*x*_Cl_1-*x*_)_3_) with various doping compositions (*x*). The Moss rule [37], $\epsilon (x)=a+b\sqrt{E_g(x)}$, is therefore adopted. The dielectric function *ϵ* is related to emission energy bandgap *E*_*g*_ of the mixed perovskites (MAPb(Br_*x*_Cl_1-*x*_)_3_) with doping composition *x*. In the formula, the dielectric function *ϵ*(*x*) of pure perovskites MAPbCl_3_ (*x* = 0) and MAPbBr_3_ (*x* = 1) at their respectively corresponding emission wavelengths 425 and 555 nm [30] was used to determine fitting constants *a* and *b*. The energy bandgap of pure perovskites were deduced from the emission wavelengths. We then obtained the energy bandgap of mixed perovskite from the relation ${E}_g^{\mathrm{MAPb}{\left({\mathrm{Br}}_x{\mathrm{Cl}}_{1-x}\right)}_3}(x)=\left(1-x\right){E}_g^{\mathrm{MAPb}{\mathrm{Cl}}_3}+x{E}_g^{\mathrm{MAPb}{\mathrm{Br}}_3}$ [38]. As shown in Fig. 2, the complex refractive index (*n*, *k*) of MAPb(Br_*x*_Cl_1-*x*_)_3_ is derived from the dielectric function, $n(x)+ ik(x)=\sqrt{\epsilon (x)}$, at each doping composition *x*. With the increase in the content of Br, doped MAPb(Br_*x*_Cl_1-*x*_)_3_ exhibits a redshifted energy bandgap and emits at longer wavelengths. The same procedure was applied on deriving (*n*, *k*) of MAPb(I_*y*_Br_1-*y*_)_3_ with I doping composition *y*, as depicted in the right section of Fig. 2. The mixture of MAPbBr_3_ (*y* = 0) and MAPbI_3_ (*y* = 1), MAPb(I_*y*_Br_1-*y*_)_3_ emits at long wavelengths from 555 to 800 nm. The refractive indices of doped perovskites are depicted in Fig. 2 and are used in the following calculations. The refractive indices of pure perovskites MAPbCl_3_, MAPbBr_3_, and MAPbI_3_ at compositions *x* = 0, *x* = 1 (*y* = 0), and *y* = 1 are (2.2, 0.013), (2.30, 0.01), and (2.49, 0.0009). They are emitting at wavelengths at 425, 555, and 800 nm, respectively.

**Fig. 3:**
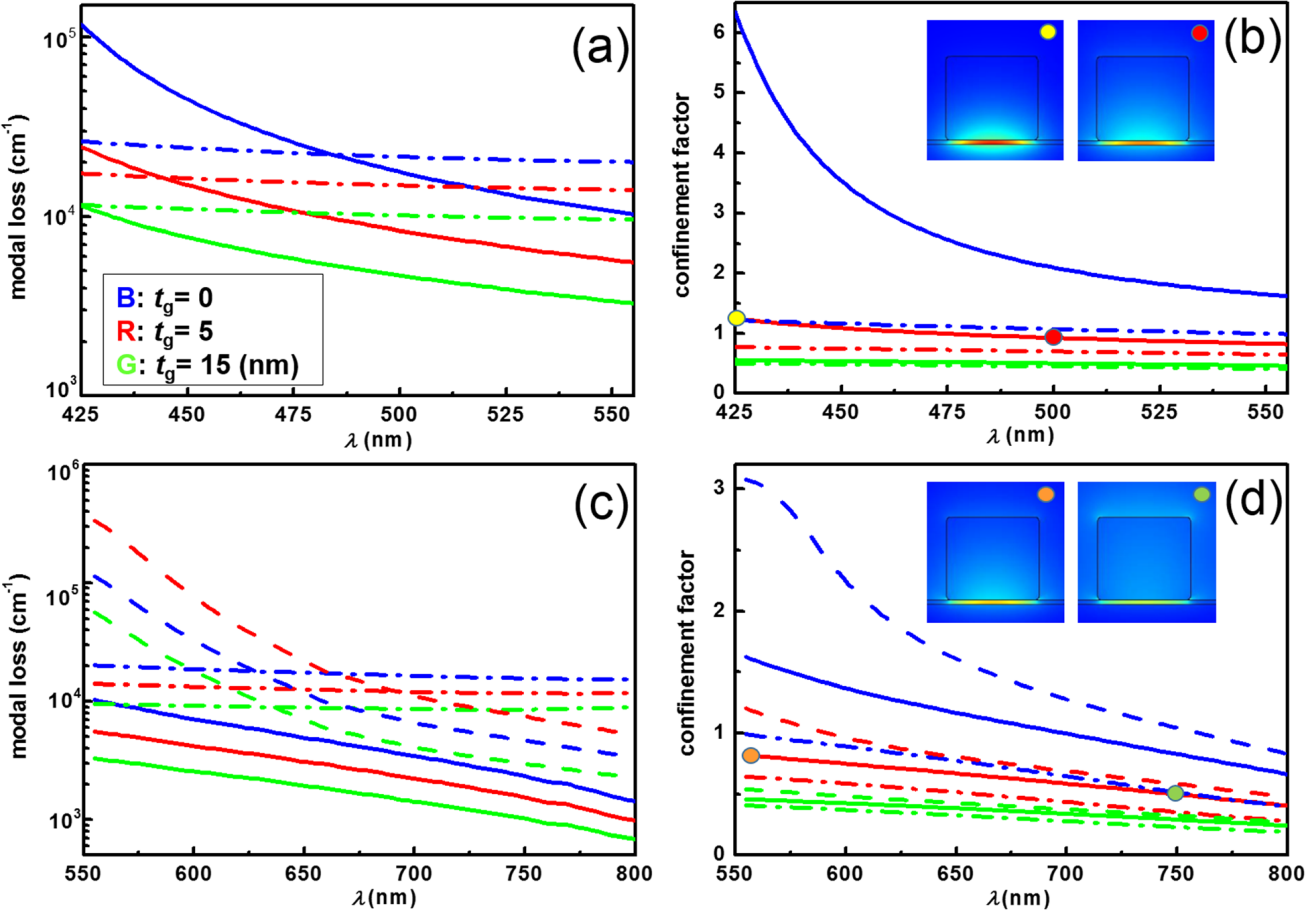
Modal loss and confinement factor of guided modes. **a**, **c** Modal loss and **b**, **d** confinement factor of the guided plasmonic gap modes at fixed SiO_2_ gap thickness, *t*_g_ = 0 (blue lines), 5 (red lines), and 15 (green lines) nm, corresponding to the doped perovskite in the photoluminescent spectrum from *λ* = 425 to 800 nm. The hybrid perovskite MAPb(Br_*x*_Cl_1-*x*_)_3_ WGs on Ag (solid lines) and Al plates (dash dotted lines) are calculated as shown in (**a**, **b**). Those of perovskite MAPb(I_*y*_Br_1-*y*_)_3_ WGs on Ag (solid lines), Al (dash dotted lines) and Au (dash lines) plates are solved at *λ* = 555 to 800 nm as shown in (**c**, **d**). The insets in (**b**, **d**) reveal the modal profiles |**E**| of the guided plasmonic gap modes on SiO_2_-covered Ag plates of *t*_g_ = 5 nm for the doped perovskites of compositions *x* = 0 (yellow circle), *x* = 0.58 (red circle), *y* = 0 (orange circle), and *y* = 0.59 (green circle)

Next, we studied the characteristics of fundamental plasmonic gap modes, which is formed by the coupling between leaky photonic guided modes (below the cutoff frequency) of perovskite NWs and surface waves concentrated mainly at the interface of gap and metal. As illustrated in Fig. 3, we determined the modal loss and confinement factor [24] of the guided hybrid plasmonic modes for the waveguide—a mixed perovskite NW, MAPb(Br_*x*_Cl_1-*x*_)_3_ and MAPb(I_*y*_Br_1-*y*_)_3_ of doping composition *x* and *y* from 0 to 1 on the SiO_2_/Ag, SiO_2_/Al or SiO_2_/Au plate of gap thickness *t*_g_ at their corresponding emission wavelengths. We determined the guided modes corresponding to a large range of emitting wavelengths, from 425 to 555 nm for the perovskite MAPb(Br_*x*_Cl_1-*x*_)_3_ and from 555 to 800 nm for MAPb(I_*y*_Br_1-*y*_)_3_. In these calculations, the complex refractive indices of the doped perovskites were (*n*, *k*) as depicted in Fig. 2. The dispersive refractive indices of metallic layers, Al, Ag, and Au, were adopted from previous experiment data [39].

With regard to perovskites emitting at wavelengths from 425 to 555 nm, the plasmonic waveguide (WG) with the NW on Al plate exhibited relatively lower modal loss (as to the Ag plate) close to short wavelengths as depicted in Fig. 3a. Thus, small metallic losses observed in the hybrid mode in the WGs on Al plates were not observed on Ag plates. One reason was that the surface plasmon frequency of perovskite/ SiO_2_/Ag was in the proximity of *λ* = 425 nm and that of perovskite/SiO_2_/Al was in the proximity of short wavelengths. The confinement of plasmonic wave near the plasmon frequency was extremely strong because of charge oscillation resonance. Therefore, the absorption of electromagnetic energy nearby was high. Otherwise, for the WG with perovskite MAPb(Br_*x*_Cl_1-*x*_)_3_ with *x* close to 1 (emitting at long wavelengths of green colors) on Al plates, modal loss can be higher than that on Ag plates. We additionally determined the confinement factor of guided plasmonic gap modes at fixed gap thickness (*t*_g_ = 0, 15, and 30 nm). The strong confinement of modal profile inside the thin gap indicated strong overlapping with the metal, thus causing severe ohmic loss. This was controlled by increasing the gap thickness. The confinement factors of perovskite WGs on Ag plates were relatively higher than other WGs on Al plates. This suggested a strong confinement of plasmonic WG modes near the gain medium on the Ag plates and a small overlap with the surroundings.

The limited overlap of guided modes with metal leads to lower modal loss as previously discussed, because metallic loss is solely responsible for the modal loss in this scheme. We can observe that, as depicted in the Fig. 3b, when the plasmon frequency of Ag approaches (around short wavelengths), the confinement factors become stronger in the WG on the Al plates. To reveal the confinement of plasmonic gap modes, we calculated the modal profiles |**E**| of a MAPb(Br_*x*_Cl_1-*x*_)_3_ NW WGs on Ag plate as depicted in insets of Fig. 3b at wavelengths of 425 (*x* = 0) and 500 nm (*x* = 0.58) at fixed *t*_g_ of 5 nm. For the WG at shorter wavelengths, or around the minimal thickness *t*_g_ = 0 nm, coupling between the nanowire photonic mode and surface plasmonic mode were stronger, leading to a highly confined plasmonic mode (as depicted in the plots with yellow circle). However, at longer emitting wavelengths of perovskite with higher doping composition, the coupling strengths become weaker. The plasmonic gap modes revealed less intensity inside the gap, and a considerable amount of energy spreads around the surrounding medium (as indicated by the picture with a red circle). The limited overlap of guided modes with metal led to lower modal loss. The tendency of the modal loss curve declined with the increase in the gap thickness. At longer wavelengths, similar to the WG with thicker gaps, lower coupling strength results in a lower strength of confinement.

**Fig. 4:**
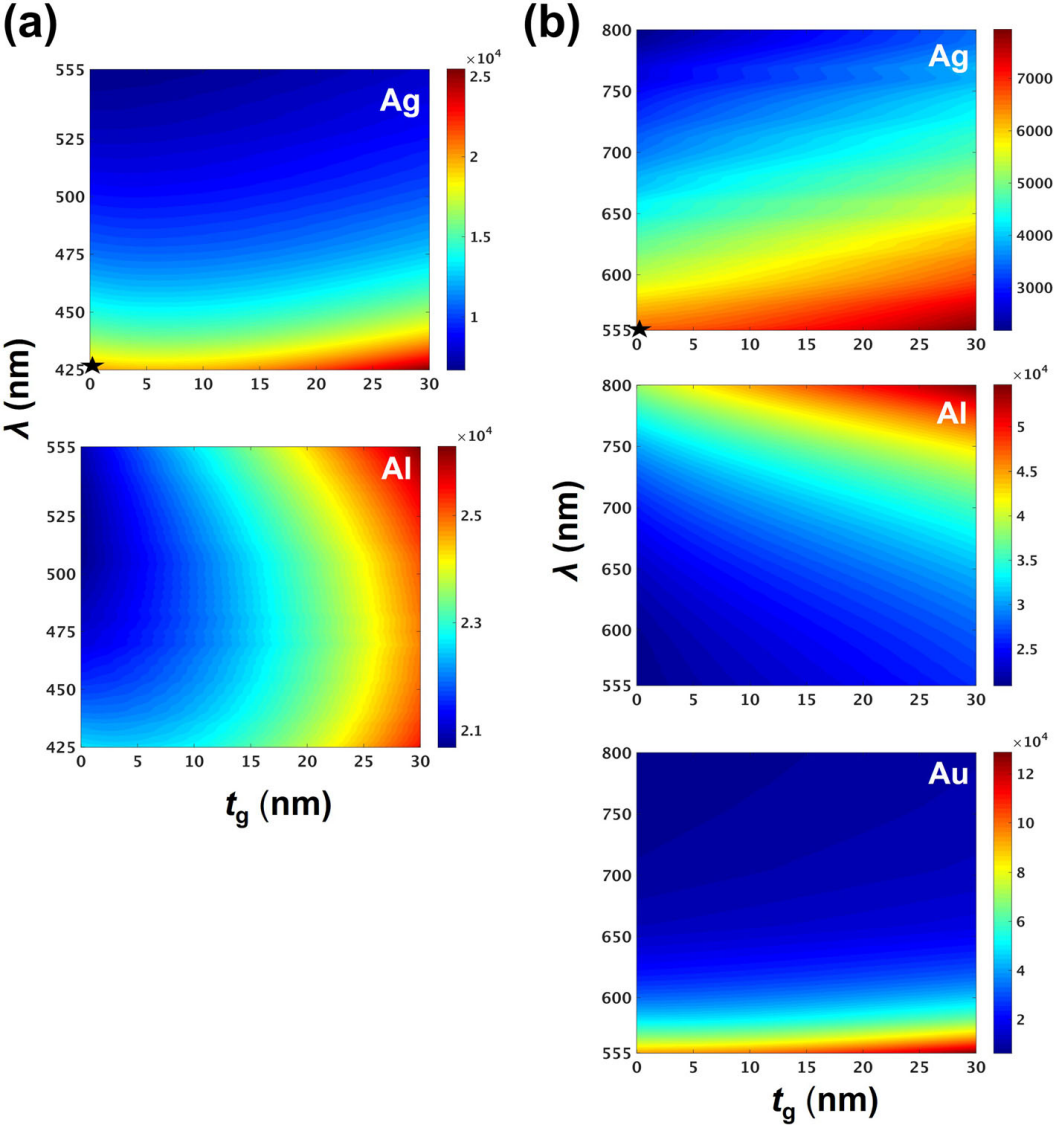
Transparency threshold gains of fundamental hybrid plasmonic gap modes. In the structures with hybrid perovskites, **a** MAPb(Br_*x*_Cl_1-*x*_)_3_ NWs on SiO_2_-coated Ag and Al plates **b** MAPb(I_*y*_Br_1-*y*_)_3_ NWs of various compositions on SiO_2_ coated Al, Ag and Au plates, corresponding to different perovskite emission wavelengths, respectively. At the minimal gap thickness *t*_g_ = 0, transparency threshold gains of the plasmonic modes on Ag plates are 18470.5 and 6259.1 denoted by the black stars in (**a**) at *λ* = 425 nm and (**b**) at *λ* = 555 nm

In WGs with hybrid perovskites emitting at wavelengths from 555 to 800 nm, the scheme with MAPb(Br_*x*_Cl_1-*x*_)_3_ NW, the Au plate may not be its suitable plasmonic medium, as inferred by the large modal loss (as to the Ag and Al plate) as depicted in Fig. 3c. The Au plate exhibited a plasmonic absorption peak at approximately 520 nm. Therefore, the intrinsic metal loss increases when approaching plasmonic wavelengths. However, superior chemical stability renders Au a preferred candidate to explore the plasmonic properties in photonic devices, especially at red and orange color wavelengths. The imaginary part of the refractive index of Ag was smaller than that of Al in this wavelength region. At approximately 550-nm wavelengths, the metal loss dominated the modal loss. Irrespective of whether the gap was thin or thick, the corresponding modal loss of Al was larger than that in Ag as depicted in Fig. 3c. Figure 3d depicted that the confinement factors of three WGs with thicker gaps are similar at longer wavelengths. The tendency of confinement factor curves and characteristics of modal profiles illustrated in Fig. 3d are affected by the coupling strengths; in a manner similar to the aforementioned discussion of Fig. 3b. To investigate the resonant modes in the cavities based on this fundamental plasmonic gap modes, which is the most likely to lase, we determined the transparency threshold gains in each case, as presented in Fig. 4.

### Threshold Performance of Plasmonic Hybrid Perovskite Nanolaser

We evaluated transparency threshold gains by using the confinement factor and modal loss of each WG for comparing resonance properties in the nanocavities of various metals and gap thicknesses. The transparency threshold is defined as the ratio of modal loss over the confinement factor [24]. As depicted in Fig. 4a, Ag exhibits superior confinement factors and transparency thresholds for each perovskite MAPb(Br_*x*_Cl_1-*x*_)_3_ WG at its corresponding emission wavelength. The optimal thickness of the cavities with the lowest threshold should be the minimal case of *t*_g_ = 0. For example, at the minimum *t*_g_ = 0, the transparency threshold gains of the plasmonic modes on Ag plates were 18470.5 and 6259.1 denoted by the black stars in Fig. 4a at *λ* = 425 nm and Fig. 4b at *λ* = 555 nm, respectively. These values were slightly lower than those at other gap thicknesses. The hybrid plasmonic mode formed by direct coupling to the surface plasmon mode exhibits ultimately confined fields. However, the modal profile suitable for the end reflectors to thoroughly reflect is often not the extremely confined profile. Furthermore, the oxidation layer is commonly formed during the deposition process, but an oxidation layer may form inexorably over time. With regard to the oxidation layer of limited thickness on the Ag plate, the threshold was relatively low when the thickness was approximately 5 to 7 nm. At the wavelengths close to 425 nm, the transparency threshold-gain of the perovskite WG on Al was slightly lower than that on Ag, as results of lower material loss and substantial overlap with the lossy region. From the discussions of modal loss and confinement factors and the results illustrated in Fig. 3, it is not difficult to anticipate the lower thresholds of the cavities on the Ag plates with doped perovskites emitting at long wavelengths of orange and red colors or infrared spectra, as depicted in Fig. 4b. The threshold was considerably high in the cavities on Au because of the relatively large material absorption. Although Al is low-cost and exhibits a limited tendency to form a measurable oxidation layer, it can still function as an excellent as a plasmonic medium in these doped perovskite-incorporated schemes because it corresponds to tolerable transparency thresholds and is less sensible to the gap thickness and doping composition, as depicted in Fig. 4a, b. Therefore, Ag is the best choice as a plasmonic medium for investigating the metal-related perovskite lasing process even though it is necessary to coat it with an oxidation layer. A low-index dielectric (oxidation layer) of approximately 5 to 10 nm thickness can sustain guided plasmonic gap modes; this gap layer may result in suitable reflection at end facets to reduce unwanted mirror loss.

After determining the spatial distribution of modal profiles as shown in Fig. 1b–d, we estimated the quality factor, *Q* using Re[*f*_*r*_]/2 Im[*f*_*r*_], where the *f*_*r*_ is the complex eigen-frequency of the resonant mode obtained using the 3D FEM. We compared these estimated values of the *Q*-factor of the resonance modes in the cavities obtained using three perovskites (MAPbX_3_; X: Cl, Br, and I) on SiO_2_-coated Ag and Al plates at a fixed gap thickness *t*_g_ of 7 nm. For fair comparison, cavity length *L* was set to four effective wavelengths (4*λ*/ Re[*n*_eff_]) at the corresponding *λ*, where Re[*n*_eff_] is the effective modal index of guided modes in each case. We concluded that because of the large intrinsic material loss of Al in the visible spectrum, the *Q*-factors of the cavities on Al plates were not comparable to Ag plates. The *Q*-factor was certainly higher in the cavity at the wavelength *λ* close to 425 nm. However, it was less capable of confining the hybrid plasmonic mode inside the gain region near the thin gaps, as indicated by the confinement factor. Therefore, the comparison of *Q*-factors also suggested that Ag is preferred in the perovskite-incorporated plasmonic scheme in the visible spectrum. Therefore, the scattering loss from end facets may not be the dominant factor that degrades the performance of cavities. As indicated by the lowest transparency threshold gains as depicted in the Fig. 4b, the resonant modes on the Ag plate close to 800 nm potentially revealed a relatively high value of *Q*-factor, which indicates potential in future applications related to plasmon-enhanced exciton-photon coupling and bio-sensing.

**Fig. 5:**
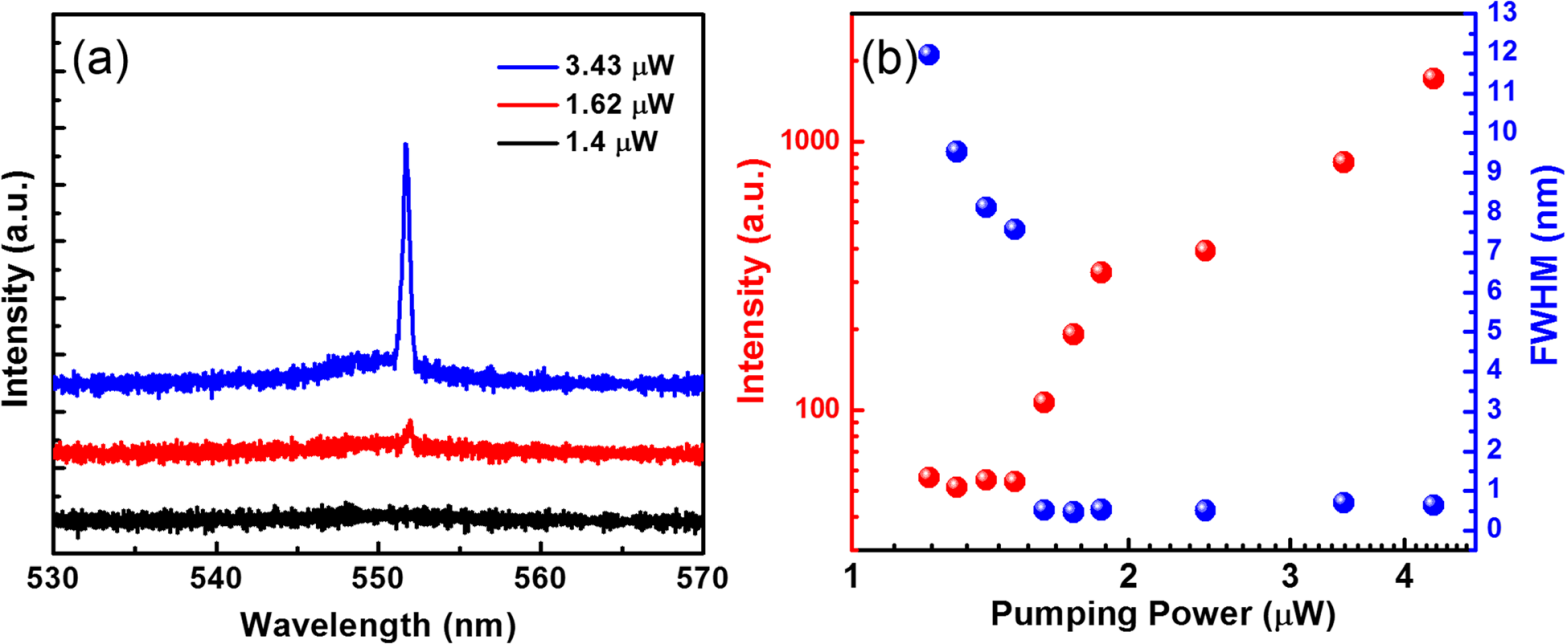
Characteristics of lasing. **a** Representative emission spectra for pumping power below (1.4 μW), near (1.62 μW), and above (3.43 μW) the lasing threshold. **b** L-L curves (red circles) and evolution of linewidths of dominant peaks with increasing pumping intensity power (blue circles) of the MAPbBr_3_ NW plasmon nanolaser on SiO_2_-covered Ag plates

Power-dependent photoluminescence was measured to resolve the emission spectra and record the lasing power at various pumping inputs, as displayed in Fig. 5. The emission spectra of the cavity with a MAPbBr_3_ NW on the SiO_2_-covered Ag plate are presented in Fig. 5a. Emission peaks in the spectrum were then fitted to obtain the light-light (L-L) curve of MAPbBr_3_ nanolaser. In the emission spectra, the output power dramatically increases at the pumping power higher than the threshold (at approximately 1.62 μW average power); the sharp change was also observed in the corresponding L-L curves as shown in Fig. 5b. Once the pumping power is higher than the lasing threshold, single-peak emission linewidth of lasing output decreases from 7.6 nm to approximately 0.5 nm. The output signals were collected from the NW end facets. The threshold power is one order of magnitude smaller than those of the ZnO NW nanolaser on the Ag plate. Possible reasons could be the superior material gain provided by MAPbBr_3_ than that of ZnO and the smaller internal loss at 550 nm than that at 370 nm [35]. Additionally, perovskite NW plasmon lasers [26–28] reveal various thresholds at different temperatures. To operate under strong pumping powers at room temperature while maintaining devices performance without severe material ablation and thermal degradation, the thermal stability [40] and crystal quality [41] of perovskite NW could be the key parameters to be improved. Desirable characteristics such as low threshold and narrow linewidth extend potential applications in future miniature active photonic devices.

## Conclusions

Full-spectrum analysis of laser parameters including guided mode characteristics, transparency threshold gains, and estimated quality factor of the perovskite-based nanolasers that featured doped perovskite nanowires placed on three types of SiO_2_-coated metallic (Ag, Al, and Au) plates was conducted. The calculated results using FEM revealed that Ag can be a suitable choice as a plasmonic metal for perovskite MAPbX_3_-based optoelectronic application. The proposed nanocavity—a MAPbBr_3_ nanowire on the SiO_2_/Ag plate, exhibited low lasing threshold and narrow linewidth corresponding to nanoscale output footprint. These advantages can result in strong coupling of exciton-polariton-photons. With the superior charge features possessed by perovskites, this scheme is an appropriate candidate for developing next-generation light sources.

## Data Availability

All data supporting the conclusions of this article are included within the article.

## Abbreviations

2D: Two-dimensional
3D: Three-dimensional
FEM: Finite-element method
L-L: Light-light
NUV: Near-ultraviolet
NW: Nanowire
SPP: Surface plasmon polaritons
WG: Waveguide
